# Supplementary material for: Isolation and characterization of Septuagintavirus; a novel clade of Escherichia coli phages within the subfamily Vequintavirinae
Source: Microbiol Spectr. 2024 Aug 5;12(9):e00592-24. doi: 10.1128/spectrum.00592-24 (PMC11370258; doi:10.1128/spectrum.00592-24)
Supplement: Supplemental figures — Fig. S1 to S3. [file spectrum.00592-24-s0001.docx]

**
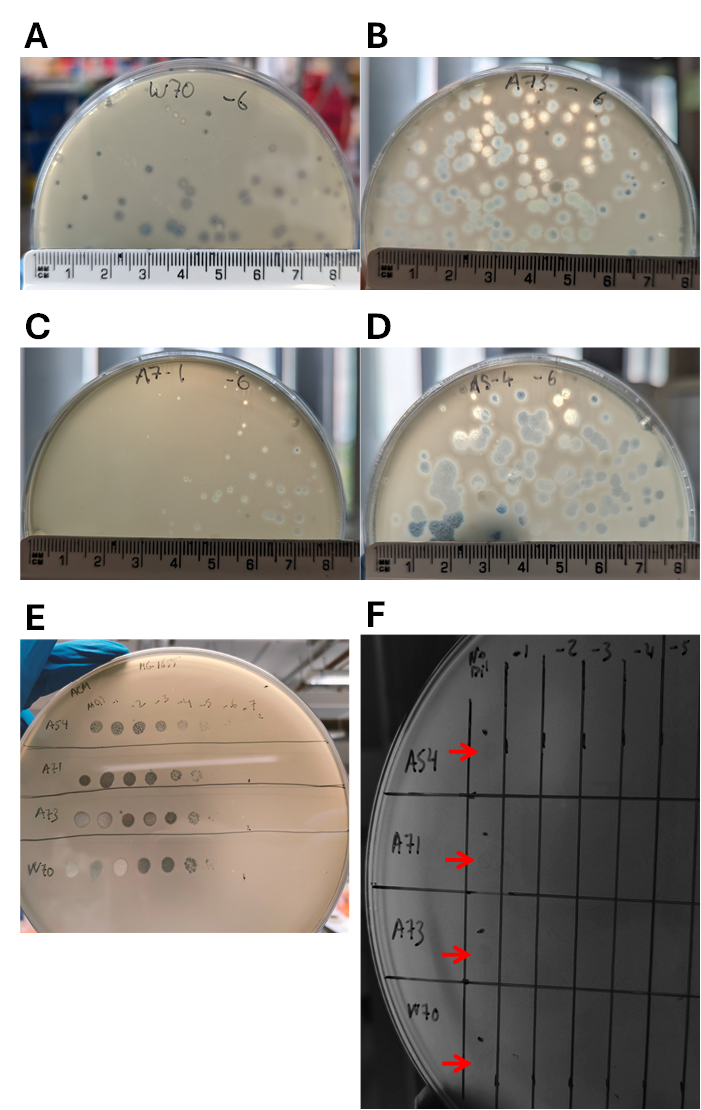
**

**Figure S1.** Plaques produced by novel isolated *E. coli* phages **(A)** W70, **(B)** A73, **(C)** A7-1, and **(D)** A5-4 when infecting the host *E. coli* K-12 MG1655. **(E)** Serial dilutions of the novel *E. coli* phages (A5-4, A7-1, A73 and W70 from top to bottom) on a plate seeded with the host *E. coli* K-12 MG1655 forming clear plaques. **(F)** Spot assay tested in the host range analysis with serial dilutions of *E. coli* phages in a plate seeded with *E. coli* 503IE-34 strain showing that is a phage-sensitive strain, producing tiny halos but no clear plaques.

**Figure S2.** W70 phage bands obtained post-purification (titre > 10^11^ pfu/mL) through **(A)** CsCl and **(B)** sucrose gradient.


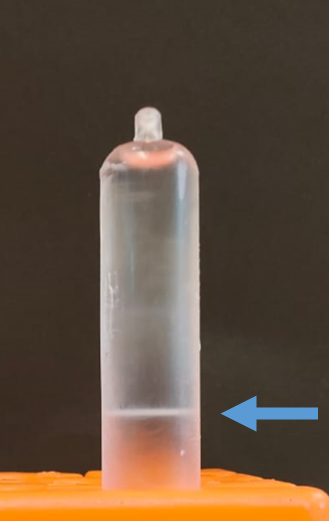

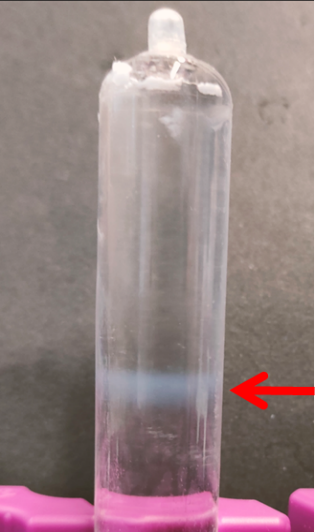


B

A

**Figure S3.** Sodium dodecyl sulphate polyacrylamide gel electrophoresis (SDS-PAGE) analysis of the purified structural W70 phage proteins (lanes A and B) on a 10% SDS-PAGE separation gel alongside an unstained protein ladder (10-200 kDa) (lane C). Peptides with higher spectrum counts that were identified using ESI-MS/MS and whose molecular weights matched the bands obtained on the gel are shown.
